# The Influence of Psychological Safety on Students’ Creativity in Project-Based Learning: The Mediating Role of Psychological Empowerment

**DOI:** 10.3389/fpsyg.2022.865123

**Published:** 2022-04-27

**Authors:** Shenghao Han, Dewen Liu, Yiliang Lv

**Affiliations:** ^1^College of Business, Shanghai University of Finance and Economics, Shanghai, China; ^2^School of Management, Nanjing University of Posts and Telecommunications, Nanjing, China; ^3^School of Management, Guizhou University of Commerce, Guiyang, China

**Keywords:** project-based learning, psychological safety, fault-tolerance culture, psychological empowerment, creativity

## Abstract

Creative-oriented new educational model will shape the direction and appearance of world development. This study focuses on the role of psychological safety and psychological empowerment in improving students’ creativity in the context of project-based learning from the perspective of student empowerment. Based on self-determination theory, we propose that psychological safety positively affects students’ creativity through psychological empowerment, and fault-tolerant culture plays a positive role in it. In this study, 238 students who participated in project-based learning were randomly selected to conduct a questionnaire survey. The results show that there is a positive correlation between psychological safety and creativity, and psychological empowerment plays an intermediary role in the relationship between them. The fault-tolerant culture enhances the direct influence of psychological safety on psychological empowerment and the indirect influence of psychological safety on creativity. Theoretical and practical implications were also discussed.

## Introduction

Creativity-centered education will shape the future world (Brazdauskas and Žirnelė, 2018). Entering the intelligent era full of dynamic and hyper-competition, creativity has become the main force to promote the sustainable development of the world, and it is also one of the most valued competencies of employers in the 21st century (Allina, 2018). Society is calling for innovative talents for higher levels of competence to address and solve environmental, social, cultural, and developmental problems (Brazdauskas and Žirnelė, 2018), and more attention is being paid to the cultivation and education of creative students than ever before. Compare with traditional education which improves learning creativity inefficiently (Hardika et al., 2018), project-based learning, as a systematic learning and research activity based on technology empowerment and teacher-student co-construction, can stimulate students’ independent inquiry and collaborative communication by creating real problem situations, and more effectively develop students’ higher-level abilities such as creativity, critical thinking ability, and entrepreneurial spirit (Anazifa and Djukri, 2017).

Although the project-based learning model has become more commoner in Chinese higher education and has an important influence on students’ psychological state and behavior, researchers have not paid enough attention to it at present. Specifically, firstly, the existing research mainly focuses on the influencing factors and internal mechanisms that affect students’ creativity in the traditional educational situation. For new-type education such as project-based learning, only experimental simulation, and popular science introduction are used to study (Solomon, 2003; Ergül and Kargın, 2014; Iwamoto et al., 2016; Anazifa and Djukri, 2017), and there is little empirical research on whether and why the project-based learning model is more conducive to enhance students’ creativity from the individual psychological and cognitive aspects. In fact, the driving factors of creativity can be divided into situational factors and individual factors (Kim and Kim, 2017). Compared with the temporary and limited influence of situational factors, individual factors can consistently and continuously influence creativity (Ahmad et al., 2022). Therefore, this paper aims to investigate the influence of individual factors of students’ creativity, so as to explore the stability and long-term antecedents of creativity promotion under the project-based learning mode. Because there are a lot of non-standardized learning contents in the course of the project, students must be liberated from the standardized and programmed indoctrination learning, and actively and efficiently carry out learning behaviors such as problem discussion, information sharing, feedback seeking, error reporting and new ideas putting forward with a relatively independent learning state. At this time, psychological safety is especially important to students. Psychological safety, as a group analysis of working environment and team as a whole (Dar et al., 2022), is the belief of individuals that it is safe to take interpersonal risks of the team (Ahmad et al., 2022). Under the uncertainty and fuzziness, the higher the psychological safety, the more likely the team members are to express different opinions and share their own knowledge, so as to enhance their creativity in freely speaking information exchange (McClintock et al., 2022; Xu et al., 2022). Therefore, this paper predicts that psychological safety can help students to make full use of the right of speaking and decision-making in project-based learning by minimizing interpersonal risks, thus enhancing creativity.

Secondly, the existing literature on empowerment under the educational background pays more attention to teachers’ empowerment, that is, empowering teachers with responsibility, choice and autonomy has a positive impact on commitment, satisfaction and trust (Kusumaningrum et al., 2019; Tindowen, 2019; Tenório et al., 2020), and lacks the analysis of students’ psychological empowerment under the new educational model. Considering that the power of teachers and students has changed greatly in project-based learning, students are empowered to lead the project, and teachers are only supervisors and guides (Iwamoto et al., 2016; Belwal et al., 2020), it is particularly important and urgent to explore the mechanism of psychological empowerment in project-based learning from the perspective of students. According to self-determination theory, satisfying people’s three basic psychological needs, namely, autonomy, competence and relatedness, is the main way to motivate people’s behavior (Good et al., 2022). Once these needs are met, it will enhance people’s autonomous motivation, promote the internalization of control motivation, and enhance their recognition of work significance and confidence in the success of the project (Dong and Wang, 2020; Luo et al., 2020). Therefore, in project-based learning, once students perceive psychological safety, they will greatly enhance their psychological needs to promote the learning process independently (autonomy needs), strengthen their confidence to solve real problems and achieve the project goals (competence needs), and gain recognition and dependence from classmates and teachers by completing a series of challenging learning activities (relatedness needs), thus enhancing students’ “active orientation and control sense” of project implementation, and enabling them to generate a higher level of psychological empowerment. With the improvement of students’ psychological empowerment level, they will have a stronger sense of responsibility and higher intrinsic motivation (Bin Saeed et al., 2019), and they are more willing to actively put forward new ideas, accept challenging learning tasks, promote the realization of innovative schemes, thereby enhancing their own creativity. Therefore, this paper predicts that psychological safety will enhance the psychological empowerment of students in project-based learning, and further promote their creativity.

In addition, all kinds of mistakes will inevitably occur in the process of innovation (Frese and Keith, 2015). To mitigate the impact of mistakes on students’ autonomy and promote students to learn from mistakes, it is necessary to cultivate a fault-tolerant culture, which can not only reduce the negative emotional impact of mistakes and the occurrence of similar mistakes in the future, but also enhance the intrinsic motivation of students’ autonomous participation, thus enhancing their creativity. Under the fault-tolerance culture, the team can face up to mistakes and provide situational support such as collaborative handling of mistakes, thus reducing the insecurity and interpersonal risks in the team. Therefore, under the culture of fault tolerance, students with high psychological safety will shift from negative emotions to error compensation and error cause analysis more quickly (Keith et al., 2021), so as to re-understand the significance of the project, improve their ability to solve problems and enhance their psychological empowerment. On the contrary, in the organizational culture where mistakes are not tolerated, people have a low sense of psychological safety, tend to hide their own problems, protect themselves too much when interacting, take defensive actions instead of speaking freely under the influence of learning anxiety, and ultimately reduce their psychological empowerment. Therefore, this paper predicts that the fault-tolerant culture can moderate the relationship between psychological safety and psychological empowerment. Overall, this study aims to answer the following research questions:

**Q1.** What is the relationship between psychological safety and students’ creativity in project-based learning?

**Q2.** Does psychological empowerment play a mediating role between psychological safety and students’ creativity?

**Q3.** Does fault-tolerant culture play a moderating role between psychological safety and psychological empowerment?

## Theoretical Background and Research Hypotheses

### Project-Based Learning and Self-Determination Theory

Project-based learning is a systematic teaching method, which requires students to explore and implement real and complex problems in a group cooperation way, and the final results need to be presented publicly (Barak and Yuan, 2021). In the process of participation, students cooperate, construct knowledge networks independently, and enhance creativity. Compare with the traditional teaching model, project-based learning covers multi-disciplinary knowledge, is student-centered, and gives students more responsibilities and powers. In the process of self-exploration, self-design, and self-execution, students can reconstruct knowledge and solve practical problems by using multi-disciplinary knowledge, instead of simply obtaining knowledge from teachers (Iwamoto et al., 2016). The teacher is only a supervisor and guide role, not too involved in the implementation of students (Belwal et al., 2020). In addition, the evaluation of project-based learning is diversified. The evaluation subject includes self-evaluation and other evaluations, and the evaluation method includes formative evaluation and summative evaluation (Anazifa and Djukri, 2017). Through the organic combination of various evaluation methods, project-based learning enables students to transfer, apply and transform into new situations based on mastering core knowledge, produce new knowledge, put it into practice, and ultimately enhance the creativity of students.

Since students in project-based learning have a considerable right and motivation to speak and make decisions through cooperation, this paper chooses the self-determination theory as the theoretical basis, research on the inner mechanism of improving students’ creativity in project-based learning. Self-determination theory is the motivation process theory of human self-determination behavior put forward by Deci and Ryan (2000), which is mainly used to explain the motivation sources behind certain behaviors of individuals. Self-determination theory holds that people’s behavior is based on different types of work motivation (autonomous motivation or control motivation). Autonomous motivation is a strong motivation tendency for people to implement behaviors based on their full recognition of the value of an activity or behavior, while controlled motivation is the motivation tendency toward the work that people are engaged in based on the external stimulation that they can bring to work (Guo and Cheng, 2021). Obviously, autonomous motivation is self-controllable and optional, and the degree of self-determination is high. However, the controlling motivation is uncontrollable and non-selectable, and the degree of self-determination is low (Chiu, 2021). According to self-determination theory, people’s behavior is the result of the combination of autonomous motivation and controlling motivation. To achieve self-determination and optimal motivation, the social environment must meet three basic psychological needs: autonomy (the ability to perceive that actions and thoughts can be freely chosen and decided, resulting in a sense of self-determination), competence (the sense of control and competence experienced by individuals when they interact effectively with the social environment), and relatedness (individuals need to keep in touch and close relationship with important others and experience a sense of belonging) (Good et al., 2022). Based on meeting these three needs, people internalize and integrate external rules and happenstance, and transform them into intrinsic motivation and self-determination, this fosters adaptability and creativity to change. Therefore, based on self-determination theory, this paper discusses whether and how psychological safety is related to creativity from the psychological and cognitive aspects of students.

### Psychological Safety and Creativity

Psychological safety is the belief that one can show and employ oneself without fear of negative consequences to one’s self-image, status, or career (Edmondson, 2018). Psychological safety describes an individual’s perception of the degree of interpersonal threat in the work environment. Specifically, individuals perform an implicit calculus at the micro behavioral decision point to assess interpersonal risks associated with behaviors such as asking questions, seeking feedback, reporting errors, or coming up with new ideas, because they may be regarded by others as ignorant, disruptive, and even incompetent (Jiang et al., 2019). A high level of psychological safety allows people to relax and think that the workplace is safe for interpersonal risk-taking and is willing to participate openly in knowledge sharing and problem-solving as the basis for innovation (Frazier et al., 2017; Jiang et al., 2019). Considering that this paper pays attention to the formation mechanism of creativity in project-based learning, which is mainly in the form of student collaboration, from the cognitive level, it is more suitable for the research background and research focus of this paper to emphasize the psychological safety of voluntary contribution and active participation within the team through minimizing interpersonal risk.

The existing researches on the influence mechanism of psychological safety mainly discuss the relationship between psychological safety and the results of innovation, creativity, communication, knowledge sharing, employee voicing behavior, and team learning in the context of enterprises (Chen et al., 2014; Newman et al., 2017), but seldom analyze the influence mechanism of psychological safety in the context of education. As creativity in project-based learning is cultivated and developed by students when they solve real-world problems independently (Hanif et al., 2019), how to reduce the inherent interpersonal threats, and promote information sharing and task coordination have become the primary problem to be solved in project promotion. Therefore, this paper hypothesizes that psychological safety may promote creativity in project-based learning for the following reasons.

First of all, in terms of information efficiency, psychological safety enables team members to voluntarily provide and make maximum use of each member’s unique information and different views (McClintock et al., 2022), and at the same time, keep their openness and active listening, to better understand the complexity of the problem and realize the reconstruction of the knowledge system (Engelsberger et al., 2021), and ultimately enhance individual creativity. Secondly, in terms of social relations, psychological safety, as an individual’s positive expectation of interpersonal consequences, can promote the accumulation of relationship-oriented social capital (Mikalef et al., 2019), optimize the effect of students’ collaboration and interaction by developing trust and reducing interpersonal risks, and stimulate the full potential of individual creativity in dynamic cooperation (Marlow et al., 2018). Finally, in terms of behavioral motivation, psychological safety helps people overcome defensive or learning anxiety (Kolbe et al., 2020), freely concentrate on productive discussion and collective goal realization, and change from self-protection inhibition motivation to intrinsic learning motivation (Chen et al., 2019), thus affecting the leap of individual creativity. Therefore, this paper puts forward the hypothesis:

**H1:** There is a positive correlation between psychological safety and individual creativity.

### Psychological Safety and Psychological Empowerment

Psychological empowerment is “an intrinsic motivation, which reflects the active orientation and sense of control over work, which is embodied in four kinds of cognition: meaning, competence, self-determination, and effect” (Prabowo et al., 2022). Among them, meaning represents the degree of fit between personal ideals, values, behaviors, and job requirements (Javed et al., 2019). Competence, which is closer to the concept of self-efficacy, reflects an individual’s belief in his ability to perform his duties or actions (Ioannidou et al., 2016). Self-determination reflects the control and autonomy of the start, adjustment, and continuous work behavior and process (Chiu, 2021). Effect reflects the degree of personal influence on the strategy, administration, operation and organizational output of work tasks (Prabowo et al., 2022). Together, these four perceptions reflect an individual’s active rather than passive orientation to a particular job role, which coincides with the fact that students are motivated and empowered to be members of a particular project team in project-based learning and need to engage in learning and problem solving on their own. At the same time, based on self-determination theory, considering that psychological safety is the view of broader interpersonal relationships and working environment, which can help individuals get more resources and support (Dar et al., 2022), this paper speculates that psychological safety may promote the promotion of psychological empowerment in project-based learning for the following reasons.

According to self-determination theory, the main way to promote work motivation is whether the external situational factors meet the three basic psychological needs of people’s autonomy, competence and relatedness (Shi et al., 2018; Luo et al., 2020). For the students who are engaged in project-based learning, they need to face and solve the practical problems of non-standard scholarship. They must break through the indoctrination and textbook-based learning process, and actively participate in the interaction with classmates and teachers with high autonomy, so as to meet the innovative goal of project-based learning. This requires students to have sufficient autonomy or control motivation in the implementation of the project, so as to encourage them to make full use of their abilities or experiences to independently complete learning and innovation (Newman et al., 2017).

In project-based learning, as an important factor for individuals to perceive external situations, psychological safety, once perceived by students, will enhance students’ intrinsic learning motivation by meeting their three basic needs, thus promoting a higher level of psychological empowerment (Good et al., 2022). Firstly, in terms of enhancing meaning, by helping individuals speak out openly and provide feedback, psychological safety promotes the good interaction between students and students, as well as between students and teachers (Liang et al., 2012), meets their relatedness needs, and helps the new value of the project be discovered and developed (Edmondson, 2018), thus improving the matching degree between individual beliefs and organizational requirements. Secondly, in terms of enhancing competence, psychological safety enhances the individual’s willingness and ability to challenge the status quo by reducing interpersonal risks (Kolbe et al., 2020), and make students believe that they can complete realistic challenging project tasks, so as to meet their competence needs, and then improves their self-efficacy. Thirdly, in terms of enhancing self-determination, psychological safety encourages people to put forward their own ideas, hold the decision-making power in their own hands, and enable students to initiate, adjust or discuss problem solutions more independently, meet their autonomy needs, so as to improve their self-determination awareness (Singh and Sarkar, 2018). Finally, in terms of enhancing effect, psychological safety helps individuals to disperse their thinking and adventurous spirit, and stimulate their exploratory learning behaviors (Lee et al., 2018), so that autonomous learning behaviors can be standardized in the organization (Newman et al., 2017), so as to meet their autonomy needs, and then the influence of individuals on the project process can be improved. Therefore, this paper puts forward the hypothesis:

**H2:** Psychological safety is positively correlated with psychological empowerment.

### Psychological Empowerment and Creativity

According to self-determination theory, the satisfaction of people’s autonomy, competence and relatedness needs in the organizational environment, will enhance people’s autonomous motivation and promote the internalization process of controlled motivation. When people have the sense of autonomy to control their behavior at work, they will have a stronger sense of responsibility and higher internal motivation (Rhee et al., 2017), and strengthen their willingness to independently implement certain activities or behaviors (Luo et al., 2020). Therefore, students with high psychological empowerment, who have such strong motivation to participate in learning, will improve their creativity at all stages from generation to realization.

First of all, in the stage of generating ideas, individuals with high psychological empowerment have more freedom to generate unique ideas, and they are more confident that their ideas will be valued in the organization (Javed et al., 2019), so it is easier to generate and display innovative ideas or solutions to tasks and problems (Abukhait et al., 2019). Secondly, in the stage of seeking support, individuals with high psychological empowerment have the opportunity to choose and take risks without fear of punishment (Khan et al., 2020), and high self-confidence and self-efficacy make the team willing to accept the inherent risks of challenging the status quo, so it is easier to get resource support based on mutual trust and cooperation (Aldabbas et al., 2021). Finally, in the stage of innovation implementation, individuals with high psychological empowerment have considerable intrinsic motivation to exert greater influence on the project implementation (Malik et al., 2021), and at the same time enjoy greater autonomy to carry out innovative behaviors in a proactive manner. Therefore, this paper puts forward the hypothesis:

**H3:** Psychological empowerment is positively correlated with individual creativity.

### The Mediating Role of Psychological Empowerment

From the perspective of self-determination theory, the high autonomy model of project-based learning can meet the three basic needs of autonomy, competence, and relatedness, enhance students’ autonomous learning motivation and behavior (Good et al., 2022), and then improve students’ creativity. Specifically, in terms of emotional support, psychological safety gives students a sense of belonging and freedom, encourages them to take on interpersonal risks and bravely express new ideas and different opinions, promotes individual members’ awareness of psychological empowerment on the basis of meeting their autonomy needs and relatedness needs, and stimulates individuals to rethink the meaning of the project and their belief in their ability to complete the project (Fernandez and Moldogaziev, 2013), thus developing their creativity. In terms of information support, psychological safety creates a free and cooperative environment of speaking freely and interacting efficiently, which stimulates students to explore and learn information and knowledge from different sources, forms the necessary conditions to enhance individual members’ psychological empowerment based on meeting the needs of competence and relatedness, and makes students feel their autonomy and influence on the construction of knowledge system and project realization (Dust et al., 2018), and then develops their creativity. Therefore, this paper puts forward the hypothesis:

**H4:** Psychological safety positively affects individual creativity through psychological empowerment, and psychological empowerment plays a mediating role between psychological safety and creativity.

### The Moderating Role of Fault-Tolerant Culture

Creativity can’t be without mistakes which can’t be completely avoided in personal development and human development (Frese and Keith, 2015). The wrong negative emotions and behaviors will seriously affect the individual’s self-efficacy (Wang et al., 2020). Given the cultural guidance behavior (Ravindra et al., 2019), this paper holds that the fault-tolerant culture will serve as an active and open organizational culture, maintain and improve students’ psychological empowerment, and ultimately guide and promote students’ creativity.

Fault-tolerant culture is a common norm, procedure, belief, and core value about facing up to the inevitability of mistakes, recognizing the input of mistakes, exchanging information and knowledge related to mistakes, and dealing with mistakes cooperatively (van Dyck et al., 2005). This organizational culture can give full play to the diversity of project-based learning evaluation to reduce negative error consequences, enhance positive results, and ultimately foster and develop students’ psychological empowerment. Specifically, on the one hand, the fault-tolerant culture can reduce the insecurity and interpersonal risks in the team, so that students with high psychological safety can quickly transfer their limited cognitive resources from negative emotions to error compensation and error cause analysis (Keith et al., 2021), thus re-understanding the significance of the project, and finally enhancing their psychological empowerment. On the other hand, the fault-tolerant culture can promote individuals to learn from failures, explore and reflect on the causes of failures, and optimize the positive effects of errors more effectively in the cognition of individuals with high psychological safety, for example, promoting more adaptive practices (Svensson de Jong, 2021), thus enhancing the autonomy and influence of individuals. On the contrary, in an environment where mistakes are not tolerated, low psychological safety will make it less likely for members to express their reflections and share their new ideas again by learning from failures, which will lead to lower self-confidence and psychological empowerment, and even a vicious circle of mistakes-low self-efficacy-repeated mistakes (Hirak et al., 2012). Therefore, this paper puts forward the hypothesis:

**H5:** Fault-tolerant culture positively moderates the relationship between psychological safety on psychological empowerment.

Moreover, in some schools, people regard mistakes as indicators of poor performance, negligence, or even lack of intelligence (Svensson de Jong, 2021), which seriously hindered the development of individual creativity. This paper holds that fault-tolerant culture will be an inclusive and open organizational culture to guide and develop students’ creative thinking and behavior. Based on the above analysis, fault-tolerant culture moderates the influence of psychological safety on psychological empowerment, while psychological empowerment plays a mediating role between psychological safety and creativity. According to this, it can be further inferred that the fault-tolerant culture may also have a moderating effect on the mediating effect of psychological empowerment between psychological safety and creativity, that is, there may be a moderated-mediation effect. Therefore, this paper puts forward the hypothesis:

**H6:** Fault-tolerant culture positively moderates the mediating role of psychological empowerment between psychological safety and creativity.

**FIGURE 1:**
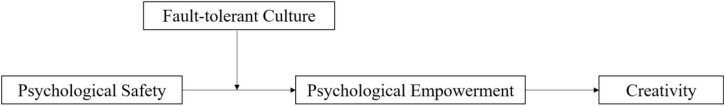
Theoretical framework. Source: Authors build this model based on relevant data.

According to the above hypothesis, the conceptual model of this paper is shown in Figure 1.

## Research Methodology

### Sample

In this study, the online questionnaire survey system^1^ was used to distribute online questionnaires to students by random sampling and collect relevant data. To reduce common method bias, respondents were told that the conditions for participation in this survey were voluntary and anonymous. Therefore, respondents do not have to guess what investigators expect, and can answer questions based on their actual situation. This study uses three filtering questions to ensure that the respondents are participants in project-based learning in higher education, that is, “What grade are you in now?,” “Have you ever experienced project-based learning in higher education?” If the answer is “yes,” the respondents are required to provide a brief description of their project-based learning experience, including the opportunity, process, and results of the experience. This method eliminates those who may accidentally join the project-based learning but are uninterested and indifferent to the project-based learning course, enhances the memory of the respondents, and improves the accuracy of the follow-up answers.

Finally, a total of 318 questionnaires were issued in the 20-day period from November 5th to November 25th, 2021. Excluding those who did not pass the filter questions, ignored the reverse questions, gave incomplete answers, and submitted the questionnaire too quickly, 238 valid questionnaires were collected, the response rate is 74.8%. Among them, 118 were males and 120 were females. Most of the respondents are undergraduate students (84.0%). The monthly living expenses mainly include less than 1,500 yuan (33.6%), 1,501–3,000 yuan (29.2%) and more than 3,000 yuan (22.6%). There are 91 students (38.2%) in soft subjects and 147 students (67.8%) in hard subjects. The demographic profile of the sample are shown in Table 1. From the perspective of the proportion of sample disciplines, it is roughly the same as that of Chinese higher education students. Judging from the other characteristics of samples, it also accords with the general characteristics of Chinese students today. Therefore, the sample is representative.

**TABLE 1:** Sample profile (*N* = 238).

	Value	Numbers	Percentage (%)
Gender	Male	118	49.58
	Female	120	50.42
Monthly living expenses (¥)	≤1500	178	74.79
	1501–3000	54	22.69
	≥3001	6	2.52
Education	Undergraduate students	200	84.03
	Postgraduate students	33	13.87
	Doctoral students	5	2.10
Subject category	Soft science^a^	91	38.24
	Hard science^b^	147	61.76
Site of the university	First-tier city^c^	25	10.50
	Second-tier city^d^	47	19.75
	Others	166	69.75
Site of hometown	First/second-tier cities	32	13.45
	Others	206	86.55

*^a^Soft science includes philosophy, economics, law, education, literature, history, and art.*

*^b^Hard science includes science, engineering, agriculture, medicine, military science, and management.*

*^c^The first-line cities represent Beijing, Shanghai, Guangzhou, and Shenzhen.*

*^d^Second-tier cities represent provincial and sub-provincial cities.*

### Measures

This paper’s questionnaire is divided into three parts: First, filtering questions. Second, the main part, which measures variables such as psychological safety, psychological empowerment, fault-tolerant culture, and creativity. Third, demographic characteristics, including gender, monthly living expenses, subject category, site of the university, site of hometown. Among them, the questions in the main part are realized by Likert scale, using the existing research maturity scale for reference. The survey was conducted in China and all items were translated in Chinese. Besides, the original items were used in work context. This paper modified some words and expressions to suit the educational context after consulting the teachers and some students.

First of all, *Psychological Safety* is measured by the six items of Edmondson (1999), including “If you make a mistake in this team, it will be bad for you” (reversed) and “It is safe to take risks in this team.” *Psychological Empowerment* is measured by Spreitzer’s (1995) classic scale, which consists of nine items, including “I have great independence and autonomy in how to study.” The dimensions of these two variables range from “strongly disagree” (1) to “strongly agree” (7).

Secondly, four items adapted by van Dyck et al. (2005) are used to measure the *Fault-tolerant Culture*, such as “Teachers and classmates will tolerate or forgive mistakes made by others in their studies.” The scale of George and Zhou (2001) is used for reference to measure *Creativity*. There are eight items, including “I often have new and creative ideas” and “I propose new ways to achieve learning goals.” The dimensions of these two variables range from “strongly disagree” (1) to “strongly agree” (5).

In addition, it should be noted that hard subject is a general term for to the cross-development of natural science and technological science. Soft subject is a group of new subjects formed by the cross development of modern natural science and social science. For this study, the difference between them lies in the degree of empowerment and autonomy of students in project learning, and the autonomy of students in soft subject is stronger than that in hard subject. Therefore, this paper holds that soft and hard subject will affect the effect of students’ psychological empowerment, which needs to be controlled.

### Common Method Bias

Since the data were collected from the same group of respondents at the same time, and all the variables were in the same environment, concerns about common method bias (CMB) became apparent (Lindell and Whitney, 2001). As a diagnostic measure, we applied Harman’s single-factor test to enter all 29 items into an unrotated principal components factor analysis to determine the number of factors required to explain the variance in the variable (Podsakoff et al., 2003). Our results suggested that there were three potential factors (all eigenvalues greater than 1) that account for 73.5% of the variance, with the highest variance explained by a single factor being 46.3% (Less than 50%), which cannot explain the majority of the differences in the study. Therefore, we concluded that CMB was not a problem in this study.

## Results

### Assessment of Measurement Models

Based on the criteria proposed by Hair et al. (2012), this study focuses on evaluating the reliability and validity of variables before evaluating the quality of structural models. Firstly, as shown in Table 2, all loadings are well above the threshold of above 0.7, indicating satisfactory indicator reliability (Bagozzi et al., 1991). In addition, the values for Cronbach’s Alpha (α) and composite reliability (CR) exceed the threshold of 0.7, indicating strong internal consistency reliability (Bagozzi and Yi, 1988). Secondly, all average variance extracted (AVE) is higher than the minimum threshold of 0.5, which indicated a high degree of convergence effectiveness (Fornell and Larcker, 1981). Thirdly, discriminant validity is evaluated based on the Fornell and Larcker criteria and the Heterofactorial-Monotrait ratio. As shown in Table 3, diagonal elements are larger than off-diagonal elements, so the square root of the AVE of each construct was higher than the correlation coefficients between constructs. Both methods have proved that discriminant validity was supported. Finally, a series of confirmatory factor combinations (CFA) are conducted to estimate the fitness of the four variables and corresponding items. As shown in Table 4, using “item parceling” method, the four-factor model indices showed that the data fit well [χ^2^(988) = 319.38, CFI = 0.951, TLI = 0.940, SRMR = 0.034, RMSEA = 0.098] and all the standardized factor loadings were greater than 0.5 significantly. What’s more, the model indices of competitive CFA models showed that the four-factor fitted the date considerably better than any of the competitive CFA models, which indicated the construct validity between the variables was qualified (Cheung and Rensvold, 2002).

**TABLE 2:** Reliability and convergent validity.

Variables	Cronbach’s α	Composite reliability	AVE
Psychological safety	0.910	0.914	0.643
Psychological empowerment	0.965	0.966	0.761
Fault-tolerant culture	0.940	0.938	0.790
Creativity	0.905	0.906	0.549

**TABLE 3:** Discriminant validity (Fornell-Larcker criterion).

Variables	1	2	3	4
1. PS	**0.802**			
2. PE	0.590***	**0.872**		
3. FC	0.184***	0.496***	**0.889**	
4. CV	0.449***	0.563***	0.502***	**0.741**
Mean	4.087	4.503	4.899	3.961
S.D.	1.284	0.986	1.038	1.078

Significant at p < 0.05 (***p < 0.001) level. PS, Psychological Safety; PE, Psychological Empowerment; FC, Fault-tolerant Culture; CV, Creativity. Bold values on the diagonal are the square root of the average variance extracted of each variable.

**TABLE 4:** Results of confirmatory factor analysis.

	χ^2^	df	CFI	TLI	SRMR	RMSEA
Four-factor model	319.38	98	0.951	0.940	0.034	0.098
Three-factor model^a^	708.57	101	0.865	0.840	0.086	0.159
Three-factor model^b^	1019.35	101	0.796	0.758	0.196	0.114
Three-factor model^c^	1057.91	101	0.787	0.747	0.137	0.143
Two-factor model^d^	1808.93	103	0.621	0.559	0.238	0.264
Single-factor model	1991.87	104	0.581	0.516	0.182	0.277

*^a^Psychological Safety and Psychological Empowerment merged as a potential factor.*

*^b^Fault-tolerant Culture and Creativity merged as a potential factor.*

*^c^Psychological Empowerment and Fault-tolerant Culture merged as a potential factor.*

*^d^Psychological Safety and Fault-tolerant Culture merged as a potential factor. Psychological Empowerment and Creativity merged as one factor.*

### Descriptive Statistics

Table 3 presents the correlations of all the variables. From the table, psychological safety was positively correlated with psychological empowerment (*r* = 0.590, *p* < 0.001) and creativity (*r* = 0.449, *p* < 0.001), psychological empowerment was positively correlated with creativity (*r* = 0.563, *p* < 0.001). This provides a basis for further hypothesis verification. According to Tsui et al. (1995), a correlation level between two variables higher than 0.75 indicates a serious multicollinearity problem. Therefore, there is no multicollinearity problem for the main variables in this study.

### Hypotheses Testing

Hierarchical multiple regression analyses were conducted to test the hypotheses. The main effect test of this paper was shown in Table 5. In terms of direct effects, since psychological safety was positively related to creativity (β = 0.391, *p* < 0.001, Model 2), H1 was accepted. Similarly, in Model 6, psychological safety was positively related to psychological empowerment (β = 0.619, *p* < 0.001), and H2 was supported. In Model 3, psychological empowerment was positively related to creativity (β = 0.481, *p* < 0.001), and H3 were supported.

**TABLE 5:** Regression analysis results.

	Creativity				Psychological empowerment			
	M_1_	M_2_	M_3_	M_4_	M_5_	M_6_	M_7_	M_8_
**Control variables**								
Gender	0.046 (0.459)	0.017 (0.185)	0.083 (0.984)	0.066 (0.786)	−0.077 (−0.675)	−0.123 (−1.343)	−0.160* (−2.009)	−0.159* (−2.264)
Monthly living expenses (¥)	0.244* (2.412)	0.201* (2.183)	0.118 (1.368)	0.123 (1.447)	0.263* (2.291)	0.194* (2.098)	0.226** (2.807)	0.234** (3.313)
Education	0.095 (0.822)	0.068 (0.642)	−0.007 (−0.074)	0.000 (0.001)	0.213 (1.622)	0.169 (1.597)	0.124 (1.348)	0.193* (2.368)
Subject category	−0.218* (−2.118)	−0.202* (−2.160)	−0.180* (−2.079)	−0.181* (−2.104)	−0.079 (−0.679)	−0.053 (−0.568)	−0.019 (−0.226)	0.030 (0.410)
Site of the university	0.137 (1.791)	0.117 (1.675)	0.065 (1.004)	0.070 (1.086)	0.150 (1.727)	0.117 (1.673)	0.116 (1.908)	0.061 (1.124)
Site of hometown	0.203 (1.388)	0.062 (0.460)	0.030 (0.245)	0.008 (0.064)	0.359* (2.164)	0.135 (0.998)	0.141 (1.207)	0.126 (1.224)
**Independent variable**								
Psychological safety		0.391*** (7.135)		0.144* (2.296)		0.619*** (11.233)	0.542*** (11.146)	0.357*** (7.396)
**Mediation**								
Psychological empowerment			0.481*** (9.871)	0.399*** (6.639)				
**Moderator**								
Fault-tolerant culture							0.383*** (8.742)	0.419*** (10.809)
**Interaction**								
Psychological safety × fault-tolerant culture								0.334*** (8.266)
Constant	−0.478 (−1.431)	−0.328 (−1.082)	−0.065 (−0.229)	−0.080 (−0.286)	−0.858* (−2.267)	−0.621* (−2.034)	−0.644* (−2.429)	−0.716** (−3.070)
*R* ^2^	0.056	0.227	0.337	0.352	0.059	0.392	0.544	0.649
Adj. *R*^2^	0.032	0.204	0.317	0.329	0.034	0.374	0.528	0.636
*F*	2.291*	9.661***	16.704***	15.546***	2.405*	21.205***	34.191***	46.918***

*p < 0.05, **p < 0.01, ***p < 0.001.

*Bracketed values in the table are standard errors.*

In terms of mediating effect, Model 4 indicted that, when both psychological safety and psychological empowerment entered the model, the regression coefficient of psychological safety became lower than Model 2 (β = 0.144, *p* < 0.05), while the regression coefficient of psychological empowerment was still significant (β = 0.399, *p* < 0.001). Thus, psychological empowerment played a partial mediating role in the relationship between psychological safety and creativity, which supported H4.

**FIGURE 2:**
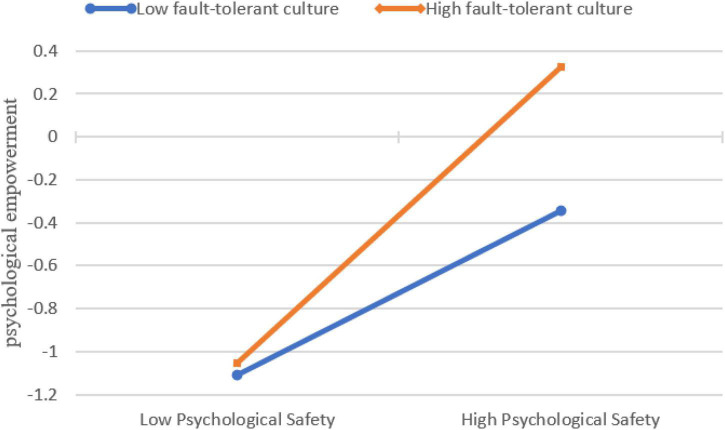
Simple slope analysis.

In terms of moderating effect, an interaction term was included in Model 8. As shown in Table 5, the interaction term between psychological safety and fault-tolerant culture was positively related to psychological empowerment (β = 0.334, *p* < 0.001), which supported H5. Simple slope analysis was performed to better show the moderating effect of fault-tolerant culture. As shown in Figure 2, when the fault-tolerant culture was low-level, psychological safety had less impact on psychological empowerment, while when the fault-tolerant culture was high-level, the relationship was strengthened. Thus, H5 was supported.

In terms of the moderated-mediation effect, the conditional indirect effect was examined to test H6 (Preacher et al., 2007). In this paper, the Process program, which involved the bootstrapping (5,000 resamplings) technique with 95% bias-corrected confidence intervals, was used to directly obtain the indirect effect when the moderator variable is low (mean−1 standard deviation), medium (mean), and high (mean+1 standard deviation), and the control variables were introduced as covariates. As shown in Table 6, the indirect effect of psychological safety on creativity through psychological empowerment varied significantly across different levels of fault-tolerant culture. The difference between the three conditions was 0.158 (95% CI = [0.035, 0.282]). Specifically, when fault-tolerant culture was of a low-level (M-1 SD), the indirect effect of psychological safety on creativity through psychological empowerment was not positively significant (Effect = 0.023, 95% CI = [−0.059, 0.101]); when fault-tolerant culture was of a high-level (Mean+1 SD), the indirect effect of psychological safety on creativity through psychological empowerment was significant (Effect = 0.262, 95% CI = [0.174, 0.342]). With the continuous increase of the level of fault-tolerant culture, the conditional indirect effect of psychological empowerment gradually increased. Thus, Hypothesis 6 was supported.

**TABLE 6:** Bootstrap analysis of moderated mediation.

	Conditional direct effect				Index of moderated mediation			
Moderator	Effect	SE	LLCI	ULCI	Effect	SE	LLCI	ULCI
Low-level	0.023	0.041	−0.059	0.101	0.158	0.063	0.035	0.282
Middle-level	0.142	0.033	0.080	0.206				
High-level	0.262	0.044	0.174	0.342				

## Discussion

### Findings

This study aims to add insights on project-based learning by exploring the mediating role of psychological empowerment in the influence of psychological safety on creativity and the moderating role of fault-tolerant culture in it.

The first research question of this paper is whether psychological safety positively affects creativity. The results show that in the context of project-based learning, psychological safety is the key factor to enhance creativity. This is consistent with the research conclusion of Javed et al. (2019) in the enterprise context, which has shown that psychological safety can support the innovative work behavior by enabling risk-taking and the willingness to suggest new ideas.

The second research question discusses the internal mechanism of psychological safety to enhance creativity. The results show that psychological empowerment plays a mediating role in the influence of psychological safety on creativity. This result reveals the significance of student empowerment in project-based learning and helps to explain why there are differences in creativity among students who both perceive psychological safety support.

The third research question discusses the boundary conditions for psychological safety to enhance creativity. The results show that the fault-tolerant culture positively moderates the positive influence of psychological safety on psychological empowerment, and then positively moderates the mediating role of psychological empowerment between psychological safety and creativity.

### Theoretical Contributions

This study provides important theoretical implications for several research streams. Firstly, this paper discusses the influence of psychological safety on students’ creativity in project-based learning, which is helpful to understand the stable influence of individual factors on students’ creativity comprehensively and deeply, it provides new empirical evidence for expanding and enriching the research on the successful mechanism of the new education model. The existing research mainly focuses on the formation mechanism of students’ creativity in the traditional educational context, and lacks empirical research on the teaching effect of new educational models such as project-based learning, this paper is the first attempt to explore whether and why psychological safety is more helpful to enhance students’ creativity in project-based learning, and expands the explanation scope and research results of psychological safety.

Secondly, based on self-determination theory, this paper discusses the mediating effect of psychological empowerment in the relationship between psychological safety and students’ creativity, and reveals the “black box” of psychological safety’s influence on students. Different from the previous literature on empowerment under the educational background, which paid attention to the empowerment of teachers (Edwards et al., 2002; Dee et al., 2003; Moye et al., 2005; Wan, 2005), this research is based on the fact that students in project-based learning have changed from passive to self-directed learning, and introduces self-determination theory, to explore the mediating role of psychological empowerment in the relationship between psychological safety and creativity. This finding not only enriches the application of self-determination theory to a certain extent, but also contributes to students’ subjective initiative and sense of responsibility in project-based learning.

Finally, this paper introduces the new concept of fault-tolerant culture, discusses the moderating variables that affect the intensity of psychological safety, and finds an important boundary condition that psychological safety affects students’ creativity. Considering the negative emotions and negative consequences caused by inevitable mistakes in innovation, this study responds to Singh and Sarkar’s (2018) discussion on the influence of organizational working environment on innovation, draws lessons from the concept of fault-tolerant culture in management, and analyses how the fault-tolerant culture affects students’ creativity by moderating the relationship between psychological safety and psychological empowerment.

### Practical Implications

These findings provide convincing enlightenment for management practice. First of all, colleges and universities that implement project-based learning and their teachers should realize that psychological safety may contribute to students’ creativity. That is to say, teachers should do some practical actions to encourage students to establish a kind of cognition and mentality to face up to interpersonal risks, dare to put forward different ideas, and make fruitful discussions in the team. For example, helping project members to share information and knowledge voluntarily and harmoniously (Iwamoto et al., 2016).

Secondly, colleges and universities and their teachers should be aware of the importance of student empowerment to enhance their creativity. Therefore, teachers need to change from classroom leaders to partners and assistants and act as catalysts, process assistants, and resources connectors when students are engaged in project learning (Hardika et al., 2018). For example, adjust the discussion rhythm and atmosphere of project team members, and provide corresponding information support and manpower support for project implementation.

Finally, colleges need to build and maintain a culture of fault tolerance, allowing team members to learn by making mistakes and accepting and thinking differences among team members (Svensson de Jong, 2021), which will help to improve students’ psychological empowerment and creativity through psychological safety. Specifically, teachers should respect and recognize students’ efforts, guide students to exchange mistakes and discuss the reasons, and follow-up compensation measures through formative evaluation, to monitor student-as-master by creating opportunities for constructive criticism.

### Limitations and Future Research

This study has several limitations, paving the way for future research. First, this study only investigates the situation of project-based learning in China, which may limit the explanatory power and universality of research conclusions. Therefore, future research can explore the adaptability of this conclusion in other cultural backgrounds and the influence of other new educational models such as problem-based learning, discovery learning, and guided inquiry on creativity.

Secondly, the results of the questionnaire survey in this study are all from students’ self-reports. Future research can directly collect students’ behavior traces and data records from schools, and conduct more accurate and comprehensive longitudinal research and investigation through actual quantitative data (such as frequency and intensity of project participation, quality, and quantity of project results).

Thirdly, the model of psychological safety affecting creativity through psychological empowerment needs further exploration. First of all, according to “The too-much-of-a-good-thing effect” (Pierce and Aguinis, 2013), the negative effects of excessive psychological safety also need to be studied and paid attention to. Moreover, this paper only considers the moderating effect of fault-tolerant culture. Among the control variables in this paper, monthly living expenses and soft and hard science have a marginally significant influence on creativity. Although they do not influence the research conclusion of this paper, they are indeed interesting phenomena worthy of attention. In the future, based on this result, the promotion mechanism of creativity in project-based learning can be further studied.

## Data Availability Statement

The raw data supporting the conclusions of this article will be made available by the authors, without undue reservation.

## Ethics Statement

Ethical review and approval was not required for the study on human participants in accordance with the local legislation and institutional requirements. Written informed consent from the patients/participants was not required to participate in this study in accordance with the national legislation and the institutional requirements.

## Author Contributions

SH contributed to conceptualization, investigation, writing, visualization, review, and editing. DL and YL contributed to the substantial revision. YL successfully applied for the sponsorship of our research. DL contributed to conceptualization, methodology, and formal analysis. All authors contributed to the article and approved the submitted version.

## Conflict of Interest

The authors declare that the research was conducted in the absence of any commercial or financial relationships that could be construed as a potential conflict of interest.

## Publisher’s Note

All claims expressed in this article are solely those of the authors and do not necessarily represent those of their affiliated organizations, or those of the publisher, the editors and the reviewers. Any product that may be evaluated in this article, or claim that may be made by its manufacturer, is not guaranteed or endorsed by the publisher.

## Footnotes

1
https://www.wjx.cn/
